# Perceived effectiveness of text-based tobacco control messages among Chinese young people: A mixed-methods study

**DOI:** 10.18332/tid/218628

**Published:** 2026-03-31

**Authors:** Yu Chen, Haoyi Liu, Zhangyan Li, Shiyu Liu, Jing Xu, Ying Yue, Kin-Sun Chan

**Affiliations:** 1 School of Art and Communication, Fujian Polytechnic Normal University, Fuzhou, China; 2 Department of Media and Communication, City University of Hong Kong, Kowloon, Hong Kong; 3 School of Media and International Culture, Zhejiang University, Hangzhou, China; 4 School of Public Health, Xi'an Jiaotong University, Xi'an, China; 5 School of Journalism and Communication, Peking University, Beijing, China; 6 China National Center for Food Safety Risk Assessment, Beijing, China; 7 Faculty of Social Sciences, University of Macau, Macau, China

**Keywords:** tobacco control, health communication, message effectiveness, young people, smoking prevention

## Abstract

**INTRODUCTION:**

Tobacco use remains a significant public health challenge among young people in China. This study evaluated the perceived effectiveness of 10 text-based tobacco control messages among Chinese young people and identified key characteristics of effective messages.

**METHODS:**

We recruited 147 young people aged 11–23 years from public schools and universities in Beijing and Kunming during March 2021. Participants completed a structured questionnaire evaluating ten tobacco control messages by selecting the single most and least effective message for preventing smoking initiation. An effectiveness index (EI) was calculated as the percentage rating each message is most effective minus the percentage rating it is least effective. Follow-up focus group interviews explored reasons for effectiveness perceptions. Quantitative data were analyzed using chi-squared tests and binary logistic regression, while qualitative data underwent thematic analysis.

**RESULTS:**

Participants had a mean age of 14.6 years (SD=2.7), with 49.0% female and 98.6% never smokers. Messages providing specific information about severe health consequences were associated with the highest perceived effectiveness. The top-ranked message detailed multiple cancer types (EI=0.285), followed by severe lung disease and COPD (EI=0.232). Three commonly used messages showed limited effectiveness: ‘No smoking, I’m healthy, I’m fashionable’ (EI= -0.470), ‘Smoking causes periodontitis’ (EI= -0.204), and ‘Refuse the first cigarette’ (EI= -0.008). Binary logistic regression confirmed that gender was significantly associated with the least effective message selection after adjusting for covariates (adjusted odds ratio, AOR=3.85; 95% CI: 1.97–7.52, p<0.001 for females selecting the ‘fashionable’ message). Qualitative analysis identified that young people valued specificity, severity, and factual information, while vague language, directive tones, and unfamiliar conditions were associated with lower perceived effectiveness.

**CONCLUSIONS:**

Tobacco prevention campaigns targeting Chinese young people may benefit from prioritizing messages providing specific, factual information about severe health risks. Messages using abstract or directive language were associated with lower perceived effectiveness, offering preliminary guidance for more effective campaigns in China.

## INTRODUCTION

Tobacco use represents one of the most pressing public health challenges globally, with young people being particularly vulnerable to smoking initiation. China, home to over 300 million smokers^1,2^, faces a critical challenge in preventing tobacco use among its young population. According to the 2019 China Youth Tobacco Survey, 19.9% of junior high school students had ever used tobacco products, and 6.9% were current users^1^. These data underscore the need for culturally appropriate prevention strategies that resonate with Chinese young people^1,2^.

Health communication campaigns serve as a cornerstone of tobacco control efforts, aiming to deter smoking initiation by raising awareness of tobacco harms and promoting anti-smoking social norms^2,3^. However, the effectiveness of these campaigns depends critically on the content and framing of their messages. Despite substantial investment in tobacco control messaging in China, systematic evaluation of which message types are most effective among young people remains limited^4^. Many current campaigns rely on messages whose effectiveness has not been empirically tested with the target population.

The Extended Parallel Process Model (EPPM) provides a theoretical framework for understanding how health threat messages influence behavior^5^. This model posits that individuals engage in two appraisals when confronted with a threatening health message: a threat appraisal (evaluating the severity of and susceptibility to the health threat) and an efficacy appraisal (evaluating the effectiveness of the recommended response and ability to perform it). Messages are most effective when they elicit both high perceived threat and high perceived efficacy, leading to danger control processes and protective action^5^.

For younger audiences, Psychological Reactance Theory provides additional insight^6,7^. This theory suggests that messages perceived as overly controlling or threatening to autonomy can trigger a boomerang effect, wherein individuals deliberately engage in the forbidden behavior to reassert their freedom. This phenomenon is particularly salient during adolescence, a developmental period characterized by heightened needs for autonomy and identity formation^8^. Traditional imperative approaches to substance use prevention, exemplified by directive messages that simply command young people to refuse tobacco, have been criticized for their simplistic, controlling nature that may inadvertently trigger such reactance^9^.

This study targets Chinese young people across primary schools, middle schools, high schools, and universities (aged 11–23 years). Although the World Health Organization defines ‘young people’ as individuals aged 10–24 years, encompassing both adolescents (10–19 years) and youth (15–24 years)^10^, our age range of 11–23 years reflects the actual age distribution of students enrolled in primary schools through universities in China during the study period. Childhood through young adulthood represents the critical window for smoking initiation, and different developmental stages may respond differently to prevention messages^11,12^. Emerging research indicates that young people respond most strongly to messages that are specific, credible, and feature severe health consequences^11,12^. However, quantitative research systematically comparing diverse message types within the Chinese context remains scarce.

This study addresses this gap by employing a mixed-methods design to: 1) quantitatively rank tobacco control messages by their perceived effectiveness among Chinese young people; 2) use thematic analysis to explore the underlying psychological mechanisms associated with these effectiveness ratings; and 3) provide preliminary recommendations for developing effective smoking initiation prevention campaigns.

## METHODS

### Study design

This study employed a concurrent mixed-methods design, combining cross-sectional quantitative surveys with qualitative focus group interviews^13^. This methodological approach was chosen to achieve triangulation, with quantitative message effectiveness rankings complementing and corroborating specific feedback from young people in interviews to identify effective message characteristics and content elements^14^.

### Participants

This study employed purposive sampling and was conducted in March 2021 in primary schools, middle schools, high schools, and universities in Beijing and Kunming. All participating institutions were public schools and universities. These two cities were selected to maximize sample diversity and enable comparison. Beijing, located in northern China, has performed relatively well in tobacco control work and has implemented smoke-free regulations compliant with the WHO Framework Convention on Tobacco Control, with relatively high public tobacco control awareness^15^. Kunming, located in southern China and the capital of Yunnan Province, represents a region where the local economy is highly dependent on tobacco, as Yunnan is a major tobacco production and consumption province with relatively low public tobacco control awareness^16^.

The target recruitment was approximately 40 participants per educational level (primary school, middle school, high school, and university), with equal representation across cities and genders within each level. A total of 147 participants were recruited from 172 eligible students, divided into 16 groups by city, school level, and gender, with 8–10 participants per group. The final sample included 35 primary school, 41 middle school, 49 high school, and 22 university participants. Inclusion criteria were: 1) currently enrolled in primary schools, middle schools, high schools, or universities in Beijing or Kunming; 2) aged 11–23 years; 3) having adequate comprehension abilities; and 4) for participants under 18 years of age, parent/guardian agreeing to participate and signing informed consent, for participants aged 18 years and older, written informed consent was obtained directly from the participants themselves. For participants under 18 years of age, written informed consent was obtained from a parent or legal guardian, and assent was obtained from the participant. The participation rate was 85.5% (147 of 172 eligible students). The study protocol received ethical approval from the Institutional Review Board of Peking University (IRB00001052-20056).

### Data collection procedures

The mixed-methods procedure followed a systematic sequential approach within each data collection session. Participants first individually reviewed all ten tobacco control messages presented in randomized order and completed quantitative evaluations using structured questionnaires. Following completion of the quantitative assessment phase, participants were organized into focus groups of 6–8 individuals stratified by city, school level, and gender. Trained facilitators conducted semi-structured interviews using a standardized guide to explore the underlying reasons for message effectiveness ratings, ensuring all participants had opportunities to contribute their perspectives on what made specific messages compelling or ineffective for tobacco prevention. This sequential approach ensured that qualitative insights could build upon and contextualize quantitative findings while avoiding potential bias from group discussions influencing individual message ratings^13^.

### Test materials

Ten text-based tobacco control messages were selected for evaluation based on a comprehensive review of existing Chinese tobacco control campaigns, international best practices, and consultation with public health experts. The messages represented three distinct categories.

*Health consequence messages (M1–M7)*

Seven messages provided factual information about specific health risks of smoking and secondhand smoke exposure^2^, including: nicotine addiction (M1), severe lung diseases and COPD (M2), periodontitis (M3), multiple cancers (M4), otitis media (M5), asthma (M6), and coronary heart disease and stroke (M7). These messages varied in the familiarity, severity, and specificity of the health conditions described.

*Directive messages (M8, M10)*

Two messages used imperative or instructive language: ‘Refuse the first cigarette’ (M8) and ‘Create a smoke-free campus, be a non-smoking new generation – please don’t tempt me to smoke’ (M10). These messages are commonly used in Chinese tobacco control campaigns and represent traditional directive approaches to prevention^9^.

*Positive framing message (M9)*

One message attempted to frame non-smoking in positive terms: ‘No smoking, I’m healthy, I’m fashionable’ (M9). This message represents attempts to associate non-smoking with desirable identity characteristics.

The complete text of all ten messages in both Chinese and English is provided in Supplementary file Table 1.

### Questionnaire content

Data collection occurred in classroom settings. The questionnaire included: 1) Demographic variables: gender (male, female), city (Beijing, Kunming), school type (primary school, middle school, high school, university), and age; 2) Behavioral assessment: current smoking status (yes, no), current e-cigarette use (yes, no), living with smoker(s) (yes, no), indoor smoking permitted at home (yes, no), and having friends who smoke (yes, no); and 3) Message effectiveness evaluation: participants selected the one message that would be most likely to prevent them from starting to smoke and the one message that would be least likely to do so. This ensured independent ratings without peer influence.

Following completion of the quantitative assessment phase, participants were organized into focus groups. Focus group discussions lasting 60–90 minutes explored participants’ reasoning for selecting certain messages as most or least effective, what elements made messages convincing or unconvincing, and their overall impressions of different message types. The facilitators explained ‘effective’ as a message that may discourage the participant from starting to smoke (indicating positive deterrent potential), and ‘ineffective’ as a message that would be unlikely to discourage smoking initiation (indicating limited deterrent potential)^11^. Discussions were audio-recorded and professionally transcribed verbatim in Chinese.

### Data analysis

*Quantitative analysis*

Descriptive statistics characterized the sample. The effectiveness index (EI) was calculated for each message as: EI = (% rating message as most effective) - (% rating message as least effective)^17^. This descriptive metric provides an intuitive measure of overall perceived effectiveness, with positive scores indicating net effectiveness and negative scores indicating net ineffectiveness. Chi-squared tests of independence examined associations between demographic variables (city, gender, school type) and message selection, with p<0.05 considered statistically significant. To assess whether demographic factors independently predicted message selection, binary logistic regression analyses were performed. To determine the most effective message, we examined whether demographic variables predicted selection of the top-ranked message (M4: cancer message) over all other messages. For the least effective message, we examined predictors of selecting M9 (fashionable message) over all other messages. Both crude (unadjusted) and adjusted models controlling for gender, city, school type, and household smoking exposure are reported. Results are presented as odds ratios (ORs) with 95% confidence intervals (CIs). All quantitative analyses were conducted using SPSS 25.0.

*Qualitative analysis*

We employed thematic analysis following Braun and Clarke’s six-phase framework^18^. Two researchers independently familiarized themselves with the data by repeatedly reading transcripts. Initial codes were generated systematically to identify features relevant to perceptions of message effectiveness. Codes were then collated into potential themes, which were reviewed against coded extracts and the entire dataset. Themes were defined and named through iterative refinement, and finally synthesized into a coherent narrative. Qualitative data were managed using Nvivo 12.0 software.

*Triangulation*

Triangulation was achieved by comparing quantitative effectiveness rankings with qualitative explanations of message characteristics, ensuring convergent validation of findings across methodological approaches.

## RESULTS

### Participant characteristics

A total of 172 participants were initially recruited, and 147 valid questionnaires were obtained after excluding incomplete responses. The final sample comprised 147 participants with a mean age of 14.6 years (SD=2.7; range 11–23 years). The sample was balanced by gender (51.0% male, 49.0% female) and relatively balanced by city (55.1% from Beijing, 44.9% from Kunming). School types were represented as follows: primary school (23.8%), middle school (27.9%), high school (33.3%), and university (15.0%). The vast majority of participants were never smokers (98.6%), with only 2 current smokers (1.4%) and 3 current electronic cigarette users (2.0%). Regarding household smoking exposure, 53.1% of participants reported living with someone who smokes, and 32.7% reported that indoor smoking was permitted in their homes. These characteristics are summarized in Table 1.

**Table 1:** Demographic and behavioral characteristics of participants in Beijing and Kunming, China, March 2021 (N=147)

*Characteristics*	*n*	*%*
**Gender**		
Male	75	51.0
Female	72	49.0
**City**		
Beijing	81	55.1
Kunming	66	44.9
**School type**		
Primary school (10–12 years)	35	23.8
Middle school (13–15 years)	41	27.9
High school (16–18 years)	49	33.3
University (19–24 years)	22	15.0
**Tobacco use behaviors**		
Current smoker	2	1.4
Current e-cigarette user	3	2.0
**Household smoking environment**		
Lives with smoker(s)	78	53.1
Indoor smoking is permitted at home	48	32.7
Has friends who smoke	52	35.4

### Overall message effectiveness rankings

Table 2 presents the rankings of perceived effectiveness. Messages providing specific information about severe, long-term health consequences were associated with the highest rankings. The message warning about multiple types of cancer (M4: ‘Smoking causes multiple cancers, including lung cancer, oral cancer, laryngeal cancer, and acute leukemia’) ranked first with an EI of 0.285, with 29.9% of participants selecting it as most effective and only 1.4% selecting it as least effective. The message about severe lung disease and COPD (M2) ranked second, with an EI of 0.232, and was selected as most effective by 25.9% and as least effective by 2.7%.

**Table 2:** Message effectiveness rankings based on effectiveness index (N=147)

*Code*	*Message content*	*Most effective* *%*	*Least effective* *%*	*EI*	*Rank*
M4	Smoking causes multiple cancers, including lung cancer, oral cancer, laryngeal cancer, and acute leukemia	29.9	1.4	0.285	1
M2	Smoking causes severe lung disease, reduced lung function, and COPD (emphysema)	25.9	2.7	0.232	2
M10	Create a smoke-free campus, be a non-smoking new generation – please don’t tempt me to smoke	14.3	7.5	0.068	3
M1	Nicotine in tobacco products is highly addictive; once addicted, it is very difficult to quit	7.5	1.4	0.061	4
M7	Smoking and secondhand smoke exposure can cause coronary heart disease and stroke	4.1	0.7	0.034	5
M6	Smoking and secondhand smoke exposure can cause asthma	0.7	0.0	0.007	6
M5	Smoking and secondhand smoke exposure can cause otitis media	0.0	0.7	-0.007	7
M8	Refuse the first cigarette	14.2	15.0	-0.008	8
M3	Smoking causes periodontitis	0.7	21.1	-0.204	9
M9	No smoking, I’m healthy, I’m fashionable	2.7	49.7	−0.470	10

EI: effectiveness index, calculated as (% Most effective) - (% Least effective). Positive values indicate net perceived effectiveness; negative values indicate net perceived ineffectiveness.

In contrast, several messages showed limited or negative effectiveness. The message ‘No smoking, I’m healthy, I’m fashionable’ (M9) ranked last with an EI of -0.470, with nearly half of all participants (49.7%) rating this message as least effective. In comparison, only 2.7% rated it as most effective. The message about periodontitis (M3) also showed low effectiveness (EI= -0.204), with 21.1% rating it as the least effective.

The widely used message ‘Refuse the first cigarette’ (M8) achieved an EI of -0.008. This near-zero score reflects the message’s polarizing nature: 14.2% of participants rated it most effective while 15.0% rated it least effective, resulting in no net positive effect. The complete rankings are presented in Table 2.

### Binary logistic regression analyses

Binary logistic regression analyses examined whether demographic factors independently predicted message selection (Table 3). For the most effective message, the adjusted model showed that female participants had higher odds of selecting the cancer message (M4) as most effective compared to males (AOR=2.31; 95% CI: 1.15–4.64, p=0.019), after controlling for city, school type, and household smoking exposure. City (Kunming vs Beijing: AOR=1.89; 95% CI: 0.94–3.81, p=0.074) and school type were not significantly associated with selection of M4 in the adjusted model.

**Table 3:** Binary logistic regression analyses of demographic predictors of message selection (N=147)

	*OR (95% CI)*	*p*	*AOR (95% CI)*	*p*
**Outcome: Selected M4 (cancer message) as most effective**				
Female (vs Male)	2.37 (1.19–4.70)	0.014	2.31 (1.15–4.64)	0.019
Kunming (vs Beijing)	1.95 (0.98–3.88)	0.057	1.89 (0.94–3.81)	0.074
Lives with smoker (vs not)	0.88 (0.44–1.75)	0.712	0.85 (0.42–1.72)	0.651
**Outcome: Selected M9 (fashionable message) as least effective**				
Female (vs Male)	4.00 (2.07–7.73)	<0.001	3.85 (1.97–7.52)	<0.001
Kunming (vs Beijing)	1.73 (0.90–3.32)	0.099	1.65 (0.85–3.21)	0.139
Lives with smoker (vs not)	1.12 (0.58–2.15)	0.734	1.08 (0.55–2.11)	0.822

AOR: adjusted odds ratio. Adjusted models control for gender, city, school type, and household smoking exposure (living with a smoker). School-type results are omitted from the table for brevity but were included in all adjusted models and were not statistically significant.

For the least effective message, the adjusted model confirmed that gender was significantly associated with selecting M9 (fashionable message) as the least effective. Female participants had markedly higher odds of selecting M9 as least effective (AOR=3.85; 95% CI: 1.97–7.52, p<0.001). City and school type were not significantly associated with M9 selection in the adjusted model. Household smoking exposure (living with a smoker) was also not a significant predictor in either model.

### Subgroup analyses

Chi-squared tests examined associations between demographic variables and message selection. As shown in Table 4, gender was significantly associated with perceptions of the least effective messages (p<0.001). Other comparisons, including city and school type, did not reach statistical significance.

**Table 4:** Subgroup analysis of message effectiveness index by city and gender in Beijing and Kunming, China, March 2021 (N=147)

*Messages*	*Beijing (N=81)*	*Kunming* *(N=66)*	*p*	*Male (N=75)*	*Female (N=72)*	*p*
M4: Cancers	0.222	0.364	0.158	0.200	0.375	0.206
M2: Lung disease	0.185	0.288	0.100	0.240	0.222	0.206
M8: Refuse first	0.012	-0.030	0.100	-0.027	0.014	<0.001*
M9: Fashionable	-0.383	-0.576	0.100	-0.293	-0.653	<0.001*
M3: Periodontitis	-0.247	-0.152	0.100	-0.333	-0.069	<0.001*

* p<0.05 indicates statistical significance.

As shown in Table 4, the significant gender difference in least effective message selection (p<0.001) was characterized by different patterns of message selection for two messages. Among females, 66.7% rated the ‘fashionable’ message (M9) as least effective (EI= -0.653), compared with 33.3% of males (EI= -0.293). Conversely, 34.7% of males rated the periodontitis message (M3) as least effective (EI= -0.333), compared with 6.9% of females (EI= -0.069). Regarding the most effective messages, 38.9% of females selected the cancer message (M4), compared with 21.3% of males; 17.3% of males selected the directive message ‘Refuse the first cigarette’ (M8), compared with 11.1% of females.

### Qualitative findings

Qualitative analysis of 16 focus group discussions identified characteristics of effective and ineffective tobacco control messages from two aspects: ‘theme and message characteristics’ and ‘presentation style’. Effective characteristics included the presentation of specific health hazards, the severity and irreversibility of consequences, and factual grounding. Ineffective characteristics included: non-specific harm presentation, directive tones, abstract language, and unfamiliar health conditions.

*Theme 1: Severity and irreversibility of health consequences*

Participants consistently expressed that messages describing severe, life-threatening health outcomes were most impactful. Cancer and lung disease messages resonated strongly because participants perceived these conditions as catastrophic, irreversible, and truly frightening. Representative quotes included:

*‘Cancer is really scary – once you get it, there’s no going back.’* (Female, High School, Beijing)

*‘Lung disease means you can’t breathe properly for the rest of your life. That’s terrifying.’* (Male, Middle School, Kunming)

In contrast, conditions perceived as less severe or more treatable failed to generate strong deterrent responses:

*‘Gum disease? You can go to the dentist for that. It’s not that serious.’* (Female, University, Beijing)

*Theme 2: Specificity and factual grounding*

Participants valued messages that provided concrete, specific information about health consequences.

Messages listing multiple cancers or describing specific disease mechanisms were perceived as more credible and informative than vague warnings:

*‘When it lists all the different cancers, it feels real and scientific.’* (Male, High School, Beijing)

Conversely, abstract or general statements were dismissed as lacking substance:

*‘ ‘‘I’m healthy, I’m fashionable’’ doesn’t tell you anything useful. It’s just empty words.’* (Female, Middle School, Kunming)

*Theme 3: Credibility and authenticity*

Young people demonstrated sophisticated evaluation of message credibility. Messages perceived as scientifically grounded and factually accurate were viewed favorably, while those seen as exaggerated, patronizing, or disconnected from reality were rejected. The ‘fashionable’ message was particularly criticized:

*‘Nobody actually talks like that. It feels like adults trying too hard to be cool.’* (Male, University, Kunming)

Scientific information about addiction mechanisms was viewed as credible:

*‘Nicotine addiction is real – I’ve seen people who can’t quit.’* (Female, High School, Beijing)

*Theme 4: Autonomy and tone*

Reactions to directive messages revealed sensitivity to perceived threats to autonomy. The ‘Refuse the first cigarette’ message polarized participants: some appreciated its directness, viewing it as protective guidance, while others perceived its imperative tone as controlling:

*‘It sounds like my parents telling me what to do. It makes me want to do the opposite.’* (Male, High School, Kunming)

*‘I think it’s good advice – if you never start, you never have to quit.’* (Female, Primary School, Beijing)

This polarization is consistent with the message’s near-zero effectiveness index.

## DISCUSSION

### Triangulation of quantitative and qualitative evidence

The integration of quantitative effectiveness rankings with qualitative explanatory insights achieved triangulation that strengthens confidence in the research findings. Quantitative data revealed patterns in message effectiveness, with health-consequence messages focusing on cancer and lung disease ranking highest. Qualitative analysis provided convergent validation by identifying the specific characteristics that young people found compelling in these same messages, including severity, specificity, and factual grounding.

This triangulation was particularly valuable for understanding why certain messages failed to achieve their intended effect. The quantitative data showed poor performance for the ‘fashionable’ message (EI= -0.470) and the directive ‘Refuse the first cigarette’ message (EI= -0.008), while qualitative insights revealed the underlying mechanisms including perceptions of inauthenticity, lack of substantive information, and inappropriate directive tone.

This mixed-methods study provides preliminary findings regarding which text-based tobacco control message strategies may be more effective among Chinese young people and, importantly, why they may be effective. The findings offer initial guidance for campaign developers while contributing to theoretical understanding of message processing among young people.

### The limited effectiveness of directive messaging

Perhaps the most notable finding of this study is the near-zero effectiveness of the ‘Refuse the first cigarette’ message (EI= -0.008). This widely used message, representative of traditional directive approaches to tobacco prevention, achieved no net positive effect among Chinese young people. The message’s polarizing nature – while some participants appreciated its directness, others reported reactions consistent with patterns described in Psychological Reactance Theory^6^ – has implications for current campaign practices in China, where this message remains prominently featured in school-based tobacco prevention programs. However, we acknowledge that the cross-sectional data and small sample size preclude definitive causal attribution of this polarization to reactance mechanisms. Alternative explanations, such as familiarity effects or perceived message relevance, may also contribute to the observed pattern.

The patterns observed among Chinese young people are noteworthy, given traditional cultural values emphasizing respect for authority. Our data suggest that contemporary Chinese young people, like their Western counterparts, may resist messages perceived as threatening their autonomy^7,8^. From a campaign effectiveness perspective, messages that may alienate substantial portions of the target audience warrant further evaluation compared to messages achieving broader consensus, such as those emphasizing severe health consequences.

### The effectiveness of specific health consequence messages

The preference for messages detailing specific, severe health consequences aligns with the threat appraisal component of the Extended Parallel Process Model (EPPM)^5^. Messages about cancer and severe lung disease may have succeeded because they established high perceived severity – outcomes universally recognized as catastrophic and life-threatening. Qualitative findings revealed that young people engaged in sophisticated evaluation of threat severity, assessing not just whether an outcome was negative, but whether it was severe enough to merit serious concern and whether it was reversible. This finding extends previous research^17,19^ by suggesting that among Chinese young people, the threshold for perceived severity sufficient to motivate deterrence may be quite high.

### The failure of aspirational messaging

The ‘No smoking, I’m healthy, I’m fashionable’ message (EI= -0.470) demonstrated the limited effectiveness of aspirational messaging among Chinese young people. This approach attempted to associate non-smoking with desirable identity characteristics but was broadly rejected. Qualitative data revealed multiple reasons for rejection: young people did not find the message credible, considered it patronizing, and criticized its lack of factual information. The significant gender difference in rejection rates (females: EI= -0.653 vs males: EI= -0.293; p<0.001) suggests that females may be particularly sensitive to perceived inauthenticity in health messaging^20^.

### Strengths and limitations

This study has several strengths. The mixed-methods design enabled both quantitative comparison across messages and qualitative exploration of underlying mechanisms. The sample spanned a wide age range (ages 11–23 years), consistent with the WHO definition of young people (ages 10–24 years), and included diverse educational levels. The inclusion of two cities with different tobacco control characteristics increased geographical diversity. The triangulation of quantitative and qualitative findings provided validation of the identified effective message characteristics.

However, several limitations warrant consideration. First, the modest sample size (n=147) limits the statistical power for subgroup analyses and the generalizability of findings. Second, the sample was drawn from only two cities in China (Beijing and Kunming) and does not capture the full range of cultural, economic, and social contexts across China’s diverse regions. As such, comparisons with international studies should be made with caution, and findings may not be generalizable to young people in other Chinese cities or provinces. Third, the sample’s predominantly never smoker composition (98.6%) means findings reflect perceptions of low-risk youth, though this aligns with our focus on smoking initiation prevention. Fourth, the cross-sectional design of the quantitative component precludes causal inferences regarding the relationship between message characteristics and effectiveness perceptions. Fifth, participants’ responses may have been influenced by social desirability bias, particularly given the classroom setting and the socially normative nature of anti-smoking attitudes among young people. Sixth, socio-economic status was not measured, which may confound message perceptions and limit the generalizability of findings. Seventh, we measured perceived effectiveness rather than actual behavioral impact, and perceived effectiveness may not directly translate to behavioral change or actual reductions in smoking initiation. Eighth, the effectiveness index (EI) is a descriptive metric that does not account for distributional uncertainty or enable formal statistical comparison across all messages simultaneously. Ninth, the exclusive focus on text-based messages limits ecological validity, as contemporary tobacco prevention messaging increasingly employs visual, multimedia, and social media formats. Future research should examine how the message characteristics identified in this study perform when integrated with visual and digital media formats that are more representative of young people’s media environments.

### Implications

These preliminary findings suggest that campaign resources may benefit from prioritizing messages that clearly communicate serious health risks, particularly cancer and lung disease. Messages should provide concrete details rather than vague warnings. Messages relying on abstract concepts without factual support, such as the ‘fashionable’ message, may warrant reconsideration. Regarding the widely used directive message ‘Refuse the first cigarette’, these exploratory findings suggest it may warrant further evaluation using larger, more representative samples before its continued use in national campaigns can be fully supported. Rather than recommending immediate discontinuation, we suggest that campaign developers consider supplementing existing messages with those emphasizing specific, severe health consequences, which showed more consistent positive perceptions in this study.

## CONCLUSIONS

This mixed-methods study suggests that Chinese young people perceive messages providing specific, factual information about severe, long-term health consequences such as cancer and lung disease as most effective for preventing smoking initiation. These messages may succeed because they establish a high perceived threat through concrete information about catastrophic, irreversible health outcomes while maintaining credibility and authenticity.

In contrast, messages showed limited perceived effectiveness when they: 1) describe less familiar or seemingly less serious health conditions; 2) use abstract language or positive framing without factual support; or 3) employ directive tones that may be associated with psychological reactance. The finding that the widely used ‘Refuse the first cigarette’ message shows no net positive effect warrants further investigation with larger samples.

To prevent smoking initiation among Chinese young people, public health campaigns may benefit from prioritizing messages that clearly communicate specific, severe health risks through factual information. Messages should inform rather than command, respecting young people’s autonomy while providing substantive reasons to avoid smoking. By aligning message strategies with young people’s communication preferences and psychological needs, practitioners may create campaigns that more effectively resonate with their target audience.

## Supplementary Material



## Data Availability

The datasets used and/or analyzed during the current study are available from the authors on reasonable request.

## References

[1] Liu S, Xiao L, Zeng X, et al. Tobacco use and exposure among secondary school students - China, 2019. CCDCW. 2020;2(22):385-393. doi:10.46234/ccdcw2020.100

[2] World Health Organization. WHO Report on the Global Tobacco Epidemic, 2021: Addressing new and emerging products; July 27, 2021. Accessed March 4, 2026. https://iris.who.int/server/api/core/bitstreams/d6747635-8c77-431e-9554-b3a2cd4d21a1/content

[3] Wakefield M, Flay B, Nichter M, Giovino G. Effects of anti-smoking advertising on youth smoking: a review. J Health Commun. 2003;8(3):229-247. doi:10.1080/10810730305686 12857653

[4] Zhao Z, Zhang M, Wu J, et al. E-cigarette use among adults in China: findings from repeated cross-sectional surveys in 2015-16 and 2018-19. Lancet Public Health. 2020;5(12):e639-e649. doi:10.1016/S2468-2667(20)30145-6 33271077

[5] Witte K. Putting the fear back into fear appeals: the extended parallel process model. Commun Monogr. 1992;59(4):329-349. doi:10.1080/03637759209376276

[6] Brehm JW. A theory of psychological reactance. Academic Press; 1966.

[7] Dillard JP, Shen L. On the nature of reactance and its role in persuasive health communication. Commun Monogr. 2005;72(2):144-168. doi:10.1080/03637750500111815

[8] Grandpre J, Alvaro EM, Burgoon M, Miller CH, Hall JR. Adolescent reactance and anti-smoking campaigns: a theoretical approach. Health Commun. 2003;15(3):349-366. doi:10.1207/S15327027HC1503_6 12788679

[9] Erceg-Hurn DM, Steed LG. Does exposure to cigarette health warnings elicit psychological reactance in smokers? J Appl Soc Psychol. 2011;41(1):219-237. doi:10.1111/j.1559-1816.2010.00710.x

[10] Adolescent health. World Health Organization. Accessed March 4, 2026. https://www.who.int/health-topics/adolescent-health#tab=tab_1

[11] Noar SM, Francis DB, Bridges C, Sontag JM, Ribisl KM, Brewer NT. The impact of strengthening cigarette pack warnings: systematic review of longitudinal observational studies. Soc Sci Med. 2016;164:118-129. doi:10.1016/j.socscimed.2016.06.011 27423739 PMC5026824

[12] Farrelly MC, Duke JC, Nonnemaker J, et al. Association between the real cost media campaign and smoking initiation among youths - United States, 2014-2016. MMWR Morb Mortal Wkly Rep. 2017;66(2):47-50. doi:10.15585/mmwr.mm6602a2 28103214 PMC5657653

[13] Creswell JW, Plano Clark VL. Designing and Conducting Mixed Methods Research. 3rd ed. SAGE Publications, Inc; 2017.

[14] Tashakkori A, Teddlie C, eds. SAGE Handbook of Mixed Methods in Social & Behavioral Research. 2nd ed. SAGE Publications, Inc; 2010.

[15] Chen Y, Liu S, Cai Y, et al. A qualitative exploration of e-cigarette prevention advertisements’ effectiveness among college students in China. Tob Induc Dis. 2024;22(June):111. doi:10.18332/tid/189300 PMC11184942 38895165

[16] He L, Cai L, Xiao X. Knowledge and current status of electronic cigarette use among college students in Kunming city. Chin Health Educ. 2021;37:820-824. doi:10.16168/j.cnki.issn.1002-9982.2021.09.011

[17] Wakefield M, Bayly M, Durkin S, et al. Smokers’ responses to television advertisements about the serious harms of tobacco use: pre-testing results from 10 low- to middle-income countries. Tob Control. 2013;22(1):24-31. doi:10.1136/tobaccocontrol-2011-050171 21994276

[18] Jensen A, Bonde LO. An arts on prescription programme: perspectives of the cultural institutions. Community Ment Health J. 2020;56(8):1473-1479. doi:10.1007/s10597-020-00591-x 32100154

[19] Zhao X, Alexander TN, Hoffman L, et al. Youth receptivity to FDA’s the real cost tobacco prevention campaign: evidence from message pretesting. J Health Commun. 2016;21(11):1153-1160. doi:10.1080/10810730.2016.1233307 27736365 PMC5101168

[20] Stalgaitis CA, Jordan JW, Isaac K. Creating more effective vape education campaigns: qualitative feedback from teens in nine U.S. States. Subst Use Misuse. 2023;58(3):406-418. doi:10.1080/10826084.2023.2165411 36621518

