# Supplementary material for: Perceived effectiveness of text-based tobacco control messages among Chinese young people: A mixed-methods study
Source: Tob Induc Dis. 2026 Mar 31;24:10.18332/tid/218628. doi: 10.18332/tid/218628 (PMC13040672; doi:10.18332/tid/218628)
Supplement: Supplementary file 1 [file TID-24-48-s1.pdf]

Supplementary file

Table 1. Complete Text of Ten Tobacco Control Messages Evaluated in Beijing and Kunming, China, March 2021

**Code**	**Chinese original**	**English translation**
M1	烟草制品中的尼古丁是高度成瘾物质，一旦成瘾很难戒断	Nicotine in tobacco products is highly addictive; once addicted, it is very difficult to quit.
M2	吸烟导致肺部严重疾病、肺功能下降及慢性阻塞性肺病（肺气肿）等多种疾病的发生	Smoking causes severe lung disease, reduced lung function, and COPD (emphysema)
M3	吸烟导致牙周炎	Smoking causes periodontitis
M4	吸烟导致多种癌症，包括肺癌、口腔癌、喉癌、急性白血病等	Smoking causes multiple cancers, including lung cancer, oral cancer, laryngeal cancer, and acute leukemia.
M5	吸烟和二手烟暴露可导致中耳炎	Smoking and secondhand smoke exposure can cause otitis media
M6	吸烟和二手烟暴露可导致哮喘	Smoking and secondhand smoke exposure can cause asthma
M7	吸烟和二手烟暴露可导致冠心病和脑卒中	Smoking and secondhand smoke exposure can cause coronary heart disease and stroke.
M8	拒吸第一支烟	Refuse the first cigarette
M9	不吸烟、我健康、我时尚	No smoking, I’m healthy, I’m fashionable
M10	创无烟校园，做不吸烟的新一代——请不要诱惑我吸烟	Create a smoke-free campus, be a non-smoking new generation – please don’t tempt me to smoke

© 2026 Chen Y. et al.
